# Melioidosis in Animals, Thailand, 2006–2010

**DOI:** 10.3201/eid1802.111347

**Published:** 2012-02

**Authors:** Direk Limmathurotsakul, Suree Thammasart, Nattachai Warrasuth, Patiporn Thapanagulsak, Anchalee Jatapai, Vanna Pengreungrojanachai, Suthatip Anun, Wacharee Joraka, Pacharee Thongkamkoon, Piangjai Saiyen, Surasakdi Wongratanacheewin, Nicholas P.J. Day, Sharon J. Peacock

**Affiliations:** Mahidol University, Bangkok, Thailand (D. Limmathurotsakul, A. Jatapai, N.P.J. Day, S.J. Peacock);; Ministry of Agriculture and Cooperatives, Bangkok (S. Thammasart, N. Warrasuth, P. Thapanagulsak, P. Thongkamkoon);; Ratchaburi Hospital, Ratchaburi, Thailand (V. Pengreungrojanachai);; Chachoengsao Hospital, Chachoengsao, Thailand (S. Anun);; Chonburi Hospital, Chonburi, Thailand (W. Joraka);; Phrachomklao Hospital, Phetchaburi, Thailand (P. Saiyen);; Khon Kaen University, Khon Kaen, Thailand (S. Wongratanacheewin);; University of Oxford, Oxford, UK (N.P.J. Day);; Addenbrooke’s Hospital, Cambridge, UK (S.J. Peacock)

**Keywords:** melioidosis, Burkholderia pseudomallei, pseudomallei, incidence, animal, human, bacteria, zoonoses, goat, Thailand

## Abstract

We retrospectively estimated the incidence of culture-proven melioidosis in animals in Thailand during 2006–2010. The highest incidence was in goats (1.63/100,000/year), followed by incidence in pigs and cattle. The estimated incidence of melioidosis in humans in a given region paralleled that of melioidosis in goats.

Melioidosis is a serious infection caused by the gram-negative bacillus and biothreat organism, *Burkholderia pseudomallei*. It is the third most frequent cause of death from infectious diseases in northeastern Thailand (after HIV/AIDS and tuberculosis) (*1*) and the most common cause of community-acquired bacteremic pneumonia in northern Australia (*2*). Melioidosis also occurs in a wide range of animal species; most cases reported in the literature are in livestock in northern Australia (*3**–**8*). In Thailand, serologic studies that use the indirect hemagglutination test (IHA) have indicated that pigs, sheep, goats, and cattle are exposed to *B. pseudomallei* (*9**,**10*), but to our knowledge, culture-confirmed melioidosis in animals has not been reported in the literature (*11*). We describe the findings of a study to estimate incidence of melioidosis in animals in Thailand and compare the geographic distribution of melioidosis in animals with that in humans.

## The Study

A retrospective study was performed to collect data on all animals recorded to have died of melioidosis and the total number of livestock in Thailand during January 1, 2006–December 31, 2010. This information was obtained from the Department of Livestock Development, Ministry of Agriculture and Cooperatives, Thailand. Information about animal melioidosis was derived from necropsies on animals that died of unknown causes which are taken to the National Institute of Animal Health in central Thailand or to 1 of 8 veterinary research and development centers throughout Thailand. Necropsy is performed principally to monitor for infectious diseases that may be associated with outbreaks in farm animals. During the study period, IHA, blood culture, and pus culture (if available) for *B. pseudomallei* were performed if melioidosis was suspected as the cause of death.

Melioidosis was diagnosed as the cause of death in 61 animals. For 49 (80%) of these animals, diagnosis was based on a culture positive for *B. pseudomallei* from >1 clinical specimens; for 12 (20%) animals, cultures were negative but samples were IHA positive for melioidosis (based on a cutoff value of >320). Cases diagnosed by IHA, but culture negative, were excluded from further analysis because apparently healthy animals in melioidosis-endemic areas can have high bacterial titers (*9**,**10*). The animal species affected were goats (31), pigs (8), cattle (4), deer (1), horse (1), and wild animals in captivity (camel, crocodile, monkey, and zebra [1 each]). Thirty-one (61%) of the 49 cases were identified during the rainy season (June–November). The estimated incidence rate of melioidosis was highest in goats (1.63/100,000/year), followed by incidence in pigs and cattle (0.02 and 0.01/100,000/year, respectively) (Table 1).

**Table 1:** Estimated incidence rates of culture-proven animal melioidosis, Thailand, 2006–2010*

Animals	Total population†	No. melioidosis cases over 5 y	Incidence rate
Goats	381,405	31	1.63
Pigs	8,187,332	8	0.02
Cattle	8,944,662	4	0.01
Others‡	NA	6	NA

*Cases/100,000 animals/year. NA, not available. †Average number of animals per year during 2006–2010. ‡One each of the following animals: camel, crocodile, deer, horse, monkey, and zebra.

We mapped the geographic distribution of melioidosis in goats by province and compared the meloidosis distribution with the total number of goats in the country during the same period (Table 2; Figure). The average number of total goats per year for 2006–2010 was 381,405. Most (41%) were in provinces in the south, with the remainder in western (19%), central (16%), northern (16%), northeastern (5%), and eastern (3%) Thailand. Although goats were not numerous in northeastern Thailand, provinces with the first and third highest incidence of goat melioidosis (Sakon Nakohn and Khon Kaen) were situated in the northeast, a region with the highest reported incidence of human melioidosis (*1*). The incidence rate of goat melioidosis was low in the south, where the incidence of human infection has not been defined but appears to be low according to cases reported in the literature (*12**–**14*). The relative incidence of goat melioidosis was also high in western and eastern Thailand, regions where human melioidosis is not considered endemic. No reports of human melioidosis in the west have been published, and the 1 report from the east described 78 blood culture–positive cases during 3 years in Sa Keao Province, from which an annual incidence rate was calculated of 4.9 per 100,000 persons (*15*). To further evaluate this finding, we contacted 4 provincial hospitals in eastern and western Thailand, where cases in animals were observed, to request information about the number of culture-confirmed melioidosis cases in humans. Culture-confirmed human melioidosis cases were observed each year in all 4 hospitals (Table 2). Our findings indicate that that the geographic area of Thailand affected by melioidosis is much greater than appreciated previously.

**Table 2:** Estimated incidence rates* of melioidosis in goats and humans, Thailand, 2006–2010

Province†	Region	Goat				Human		
		Total population‡	No. cases over 5 y	Incidence rate		Total population§	No. cases over 5 y	Incidence rate
Sakon Nakhon	Northeast	397	2	100.76		1,115,900	1,082	19.39
Ratchaburi	West	9,027	16	35.45		834,107	50	1.20
Khon Kaen	Northeast	1,372	1	29.15		1,757,772	2,122	24.14
Chachoengsao	East	1,433	2	27.91		664,184	151	4.55
Chonburi	East	2,106	1	9.50		1,262,661	76	1.20
Songkhla	South	18,355	4	4.36		1,335,832	Unknown	Unknown
Trang	South	7,058	1	2.83		614,797	Unknown	Unknown
Phetchaburi	West	7,823	1	2.56		459,398	8	0.35
Nakhon Si Thammarat	South	10,009	1	2.00		1,513,936	Unknown	Unknown
Patthani	South	19,768	1	1.01		643,718	Unknown	Unknown
Yala	South	33,325	1	0.60		476,437	Unknown	Unknown

*Per 100,000 cases/year. †Provinces are ordered by incidence rate of goat melioidosis. ‡Average number of goats per year during 2006–2010. §Average human population per year during 2006–2010.

**Figure:**
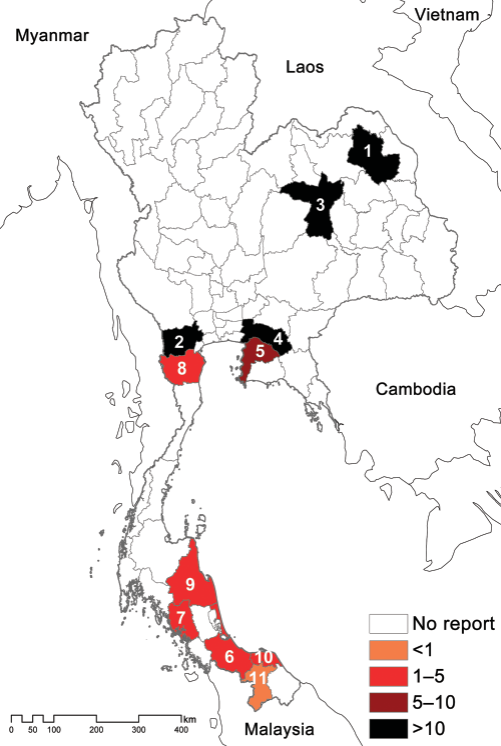
Map of estimated incidence rates for goat melioidosis, Thailand, 2006–2010. Provincial codes: 1, Sakon Nakhon; 2, Ratchaburi; 3, Khon Kaen; 4, Chachoengsao; 5, Chonburi; 6, Songkhla; 7, Trang; 8, Phetchaburi; 9, Nakhon Si Thammarat; 10, Patthani; 11, Yala. Provinces are ordered by estimated incidence of goat melioidosis.

## Conclusions

The cases of animal melioidosis in Thailand reported here probably underestimate actual cases because necropsies are performed on a small minority of animals that die of natural causes. Goats are a major domestic animal in Thailand, particularly in western and southern Thailand. We demonstrated that the estimated incidence of melioidosis in goats relative to other animals is high and might represent a substantial cause of economic loss for goat farmers. The high susceptibility of goats to melioidosis relative to that of pigs and cattle is consistent with a previous report from Australia (*5*). Mapping of goat melioidosis demonstrated concentrations of cases in specific provinces and large areas of the country with no apparent disease. This finding might represent an artifact, based on the availability of information from specific veterinary centers, and we propose that animal melioidosis might be underestimated and widely distributed across the country. We predicted that the incidence of melioidosis in goats would parallel that in humans because both would be exposed to similar levels of environmental *B. pseudomallei*. This finding proved to be the case in northeastern Thailand, where the incidence of human melioidosis is high and where a small population of goats also had a high relative incidence. Such was not the case in the west, however, where the relative incidence of goat melioidosis was high, but human cases have not been reported in the literature. Possible explanations include a more active approach to determine causes of death in goats or failure to diagnose or report human disease in the west and, less plausibly, the presence of an animal-adapted *B. pseudomallei* strain that fails to infect humans. Contact with hospitals in western and eastern Thailand indicated that melioidosis was more common than the literature suggests. Further studies to determine an accurate incidence of human and animal melioidosis throughout Thailand are required. The ability to culture and detect *B. pseudomallei* in clinical microbiology laboratories countrywide also needs to be improved (*1**,**15*).

Our findings support the recommendation for pasteurization of goat milk before consumption in Thailand, which is necessary to prevent human brucellosis and also will prevent ingestion of live *B. pseudomallei* in milk. Our study was not designed to detect evidence for animal-to-human transmission in potentially at-risk populations, such as herdsmen, veterinarians, or abattoir workers. Such detection is a future objective in Thailand because presumptive zoonotic infection has been reported in Australia (*5*). Melioidosis is not currently part of the animal disease control program in Thailand, but its inclusion may now warrant review.
